# Alitretinoin in the treatment of cutaneous T‐cell lymphoma

**DOI:** 10.1002/cam4.4237

**Published:** 2021-08-25

**Authors:** Till Kaemmerer, Pia‐Charlotte Stadler, Leonie Helene Frommherz, Anne Guertler, Lars Einar French, Markus Reinholz

**Affiliations:** ^1^ Department of Dermatology and Allergy University Hospital LMU Munich Germany; ^2^ Dr. Phillip Frost Department of Dermatology & Cutaneous Surgery Miller School of Medicine University of Miami Miami Florida USA

**Keywords:** cancer management, drug discovery and delivery, non‐Hodgkin's lymphoma, survival

## Abstract

**Introduction:**

In this survey, we analyzed data from patients suffering from the most common cutaneous T‐cell lymphomas (CTCLs) subtypes mycosis fungoides (MF) and Sézary syndrome (SS), treated with the retinoid alitretinoin during a 7‐year period at our outpatient department between 2015 and 2020.

**Materials and Methods:**

We analyzed patient medical records including TNMB stage, side effects under therapy with alitretinoin, time to next treatment (TTNT), and previous photo documentation.

**Results:**

A total of 35 patients with MF (*n* = 28) and SS (*n* = 7) were included in the study, of whom 69% were male and 31% were female. The mean age of onset was 56 ± 15 years in MF and 65.4 ± 10.8 years in SS with 51.4% having early stage (IA–IIA) and 48.6% having advanced stage (IIB–IVA) CTCL.

Of these patients 37.2% responded to alitretinoin, 28.6% had a stable course, and 34.3% experienced progression. Alitretinoin was administered as a monotherapy (25.7%) or combined with five concomitant therapies (74.2%), most frequently with ECP (31.4%) and PUVA (11.4%). 63% did not report any side effects, most often hypertriglyceridemia (20%) was described.

**Conclusion:**

Considering that nearly two thirds of the CTCL patients treated with alitretinoin showed a response or stable disease, together with a low number of side effects and low cost compared to bexarotene, alitretinoin may be a potential alternative in the treatment of less advanced CTCLs. This survey represents the largest number of recorded therapies with the retinoid alitretinoin in CTCLs in a European patient collective.

## Abstract 2

We analyzed data from patients suffering from the CTCL subtypes mycosis fungoides (MF) and Sézary syndrome (SS), treated with the retinoid alitretinoin during a 7‐year period at our outpatient department. Together with a low number of side effects and low cost compared to bexarotene, alitretinoin may be a potential alternative in the treatment of less advanced CTCLs.

## 1 INTRODUCTION

Cutaneous T‐cell lymphomas (CTCLs) belong to the group of extranodal non‐Hodgkin lymphoma, characterized by neoplastic T‐cell infiltration into the skin. Its’ incidence is estimated at 0.5/100.000 new cases per year with men being more often affected than women (1.6:1–2:1).^1^, ^2^ The average age at disease onset is 55–60 years. CTCLs can present with a variety of symptoms such as erythematous patches and plaques in earlier stages and nodal tumor infiltration in later stages of disease. The most common variants of CTCLs are mycosis fungoides (MF) and Sézary syndrome (SS).^3^

MF initially manifests as erythematous patches, plaques, and skin tumors with potential disease progression and involvement of blood, lymph nodes, and more rarely other organs. Due to its similarity to benign skin diseases like eczema, atopic dermatitis, and psoriasis in early stages of disease, diagnosis is complicated and often delayed for many years.^4^, ^5^, ^6^ In the slowly progressive non‐aggressive form, 80% of patients have a normal life expectancy. However, a minority of MF patients experience disease progression with lymph node involvement and skin tumors.^2^ SS presents itself clinically with erythroderma and is considered to be a leukemic form with malignant lymphocytes in the blood and the lymph nodes. It is characterized by a high rate of resistance to therapy with a low survival rate of less than 3 years after diagnosis.^7^ As there is currently no curative therapy approach for MF and SS available, the therapy focuses on symptom relief and reduction of disease progression. In early stages (stages IA, IB, IIA), local topical corticosteroids (TCS) and narrow‐band ultraviolet B radiation (UVB) are standard of care. As the disease progresses into a more advanced stage, additional systemic therapies are used in Europe such as extracorporeal photopheresis (ECP), psoralen and ultraviolet A (PUVA), interferon, radiotherapy, and retinoids.^8^ Nevertheless, in advanced stages, all therapies remain limited to slowing disease progression.^9^ Retinoids are small, lipophilic molecules related to vitamin A. They are used in dermatology for both topical and systemic therapy. Retinoids are most frequently used in the treatment of severe acne, various forms of psoriasis, and chronic hand eczema, but also in the treatment or prevention of certain tumor diseases of the skin like advanced stages of CTCLs. The exact mechanism of action of retinoids has not yet been fully elucidated. The effect of the activation of two distinct families of nuclear receptors, which regulate gene transcription, is discussed: selective retinoic acid receptors (RARs) and retinoic X receptors (RXRs).^10^ For example, isotretinoin and acitretin as first retinoids are used for the treatment of CTCLs, bind RARs, leading to immunomodulatory effects by promoting CD81 T‐cell responses and upregulation of Langerhans cell antigen presentation.^11^, ^12^ In the following years, it became apparent that the activation of RXRs might be of additional benefit in the treatment of CTCLs by induction of apoptosis through the caspase pathway and activation of p53.^13^, ^14^ This resulted in the development of bexarotene, which binds specifically to RXR. Currently, bexarotene is the only retinoid licensed for the treatment of CTCLs and shows good results in early and advanced stages of CTCLs.^15^, ^16^ Nevertheless, efficacy is accompanied by significant side effects, especially at higher doses, such as hypertriglyceridaemia and hypothyroidism, which may require a reduction or interruption of therapy.^17^ Also, bexarotene is not available in all markets (e.g., Canada), leading to the need for alternative treatment options.^18^ Alitretinoin binds both RARs and RXRs and is known to have a more favorable safety profile compared with other vitamin A derivatives regarding mucocutaneous side effects and impairment of laboratory findings of serum cholesterol, triglycerides, and thyroid parameters.^10^ Several case reports, a small prospective investigation, and a nationwide retrospective review showed promising results with a low incidence of side effects of the retinoid alitretinoin in the treatment of CTCLs.^18^, ^19^, ^20^ However, further studies are needed to demonstrate the effectiveness and safety of alitretinoin in the treatment of MF and SS. In this study, we analyze and summarize the clinical characteristics under therapy of alitretinoin alone or in combination with other standard therapies in a cohort of 35 patients with diagnosis of MF and SS presenting in our outpatient clinic between 2015 and 2020. We focus on disease classification, staging, epidemiological factors, effect of therapy, development of side effects, and duration of therapy.

## 2 MATERIALS AND METHODS

### 2.1 Data acquisition

After receiving ethical approval, retrospective patient data were collected using electronic records, containing patients’ medical history, laboratory results, and assessment of photo documentation. Retrieved data for each patient were TNMB staging, treatment modalities, duration of treatment, and side effects while treated with alitretinoin.

### 2.2 Selection of patients and staging

Thirty‐five patients were analyzed, who received therapy with alitretinoin (10–30 mg daily) upon confirmed diagnosis of MF (*n* = 28) and SS (*n* = 7) between 2015 and 2020 in our outpatient department. Eight patients started and continued treatment before 2015, therefore we also included them in the study. The retrospective staging of TNMB occurred according to the classification by ISCL/EORTC from 2007. Patients underwent a physical examination, complete blood cell count with examination for Sézary cells, a general chemistry panel, skin biopsy, immunohistochemistry, and T‐cell clonality testing. Patients with palpable lymph nodes underwent nodal ultrasound. In patients with advanced skin involvement or suspicious lymph nodes additional imaging was conducted. The skin biopsies were classified according to ISCL with superficial lymphoid infiltrate with epidermotropism and/or atypia.

### 2.3 Therapy evaluation

In order to assess the course of therapy with alitretinoin, a randomized assessment of the past photo documentation of each patient was performed by three independent physicians (1200 images in total). Of the 35 patients, photo evaluation was not possible for 11 patients. A classification of the previous therapies and duration until the initiation of alitretinoin was made. To examine the effect of alitretinoin, we assessed the response rate at the earliest after 4 weeks of therapy. Furthermore, disease progression was evaluated by means of time to next treatment (TTNT), whereby the change in therapy from alitretinoin to another drug was considered as a disease progression or non‐response to therapy. Possible outcomes of the therapy were set as complete response (CR), partial response (PR), stable disease (SD), and progressive disease (PD). According to the ISCL, we defined CR as full clinical remission of the skin lesions, PR as 50%–99% reduction of skin lesions from the baseline, SD as <25% increase to <50% remission in skin lesions from the baseline, and PD as ≥25% increase of skin lesions from the baseline or new tumors.^21^ To assess side effects, we evaluated the laboratory chemistry results, taken every 4–8 weeks, including cholesterol, triglycerides, and thyroid parameters. Elevated serum cholesterol was defined on the basis of data from previous trials on alitretinoin in CTCLs as values above 300 mg/dl (7.77 mmol/L) and high serum triglyceride values above 500 mg/dl (5.66 mmol/L).^20^ Fasting lipid parameters were not available for all patients and all visits. With only a small number of patients, we decided to limit the statistical analysis to descriptive percentage rates only, to estimate a trend.

### 2.4 Statistical analysis

To analyze the available data of 35 patients, a table with 11 parameters was compiled using the statistics software IBM SPSS Statistics for Windows, Version 26.0 (IBM Corporation). Each dataset was numerically encoded to allow statistical analysis. Patient data were evaluated anonymously. Descriptive data were represented by absolute and relative frequency, additionally in part by mean value, median, and standard deviation. Using the descriptive data, frequency tables and graphs were generated. To investigate the interrelations between two variables, cross tables were used and absolute and relative frequency for the individual subgroups were determined. Via chi‐square test two features were tested for independence.

## 3 RESULTS

We retrospectively analyzed the electronic charts of 35 patients with MF (*n* = 28) and SS (*n* = 7), who received treatment with oral alitretinoin (10–30 mg daily) as a monotherapy or in combination with additional standard therapies. The treatment was carried out after individual assessment as an off label application. At our center, topical corticosteroids (TCS) are part of baseline therapy for CTCLs, other therapies being an add‐on, which is why these are not listed separately.

### 3.1 Patient demographics

In our study, 68.6% (*n* = 24) of the patients suffering from MF/SS were men and 31.4% (*n* = 11) were women. The mean age at diagnosis was 56 ± 15 years (range 26–77 years) in MF and 65.4 ± 10.8 years (range 49–79 years) in SS. 51.4% (*n* = 18) had an early disease stage (IA: 14.3%; IB: 37.1%) and 48.6% (*n* = 17) had an advanced stage (IIB: 17.1%; IIIA: 8.6%; IIIB: 3.6%; IVA1: 20%) CTCL as shown in Table 1.

**TABLE 1:** Summary of demographic and clinical staging characteristics according to ISCL/EORTC classification

	Overall	Mycosis fungoides (MF)	Sézary syndrome (SS)
	*n* = 35	*n* = 28	*n* = 7
Age (years)		56 (26–77)	65 (49–79)
Stages (TNMB)			
I			
IA	5 (14.3%)	5 (17.9%)	0
IB	13 (37.1%)	13 (46.4%)	0
II			
IIA	0	0	0
IIB	6 (17.1%)	6 (21.4%)	0
III			
IIIA	3 (8.6%)	3 (10.7%)	0
IIIB	1 (3%)	1 (3.6%)	0
IV			
IVA1	7 (20%)	0	7 (100%)

### 3.2 Treatment conditions

**FIGURE 1:**
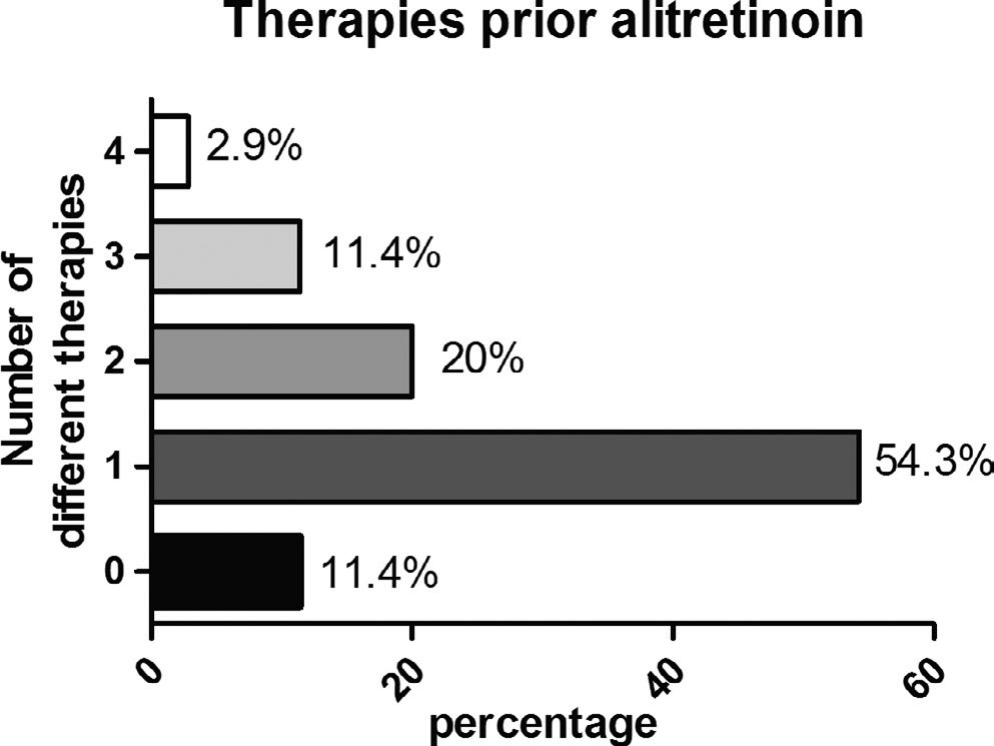
Therapies prior alitretinoin. Amount of therapies before initiation of alitretinoin in months

The median time after diagnosis, at which alitretinoin was initiated (TTA), was 14 months (mean time 44 months, range 1–288 months). On average, one systemic therapy was given before starting oral alitretinoin (range 0–4) (Figure 1). As shown in Table 2, 74.3% (*n* = 26) received alitretinoin in combination with concomitant standard therapies and 25.7% (*n* = 9) received monotherapy with alitretinoin. On average, patients received five different concomitant therapies in addition to alitretinoin, most commonly ECP (31.4%), followed by PUVA (11.4%), INF‐α (8.6%), UVB (8.6%), ECP with PUVA (8.6%), and PUVA with radiotherapy (5.7%). The mean treatment duration with alitretinoin alone or in combination with concomitant therapies was 24.5 months (range 3–83 months) for MF and 11.1 months (range 2–23 months) for SS.

**TABLE 2:** Distribution of alitretinoin with different concomitant therapies in percentages

	Overall	Mycosis fungoides (MF)	Sézary syndrome (SS)
	*n* = 35	*n* = 28	*n* = 7
Single‐agent alitretinoin	9 (25.7%)	9 (32.1%)	0
Combinations with alitretinoin			
INF‐a	3 (8.6%)	3 (10.7%)	0
ECP	11 (31.4%)	8 (13.8%)	3 (42.9%)
PUVA	4 (11.4%)	3 (10.7%)	1 (12.5%)
UVB	3 (8.6%)	2 (7.1%)	1 (12.5%)
PUVA + Radiotherapy	2 (5.7%)	2 (7.1%)	0
PUVA + ECP	3 (8.6%)	1 (3.6%)	2 (28.6%)

### 3.3 Response

**FIGURE 2:**
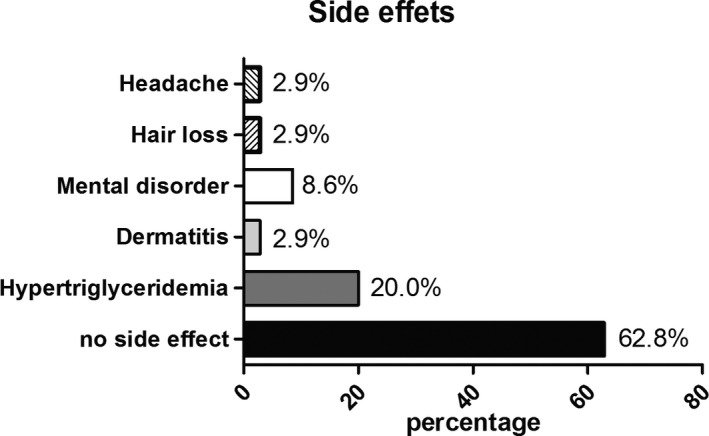
41‐year‐old man with MF (IB) (A) Initial skin findings. (B) Skin findings after 8 months of therapy with alitretinoin 30 mg daily

In 8.6% of cases (*n* = 3/35), a complete response (CR) of skin findings occurred. Of those three patients, all had MF, predominantly in early stages (IA: 1; IB: 1; IIIB: 1). 28.6% (*n* = 10/35) had a partial response (PR), of which nine patients had MF (Figure 2) and one patient had SS. Of these partial responders, seven patients had early stage disease (IA: 3; IB: 4) and three patients had advanced stage disease (IIB: 1; IIIA: 1; IVA1: 1). These values add up to an overall response rate (ORR) of 37.2% (*n* = 13/35). 28.6% (*n* = 10/35) had stable disease (SD), whereof eight patients had MF and two patients had SS. Six patients had early stage disease (IB: 1; IIA: 5) and four patients had advanced stage disease (IIB: 2; IIIA: 1; IVA1: 1). 34.3% (*n* = 12/35) developed a disease progression (PD), of whom eight patients had MF and four patients had SS with four patients in early stage disease (IA: 1; IB: 3), and eight patients in advanced stage disease (IIB: 3; IIIA: 1; IIIB: 3; IVA1: 1). For further information see Table 3. Comparing the response rate with the different therapeutic approaches, CR shows the overall dominance of alitretinoin in combination with ECP (*n* = 2/3), followed by alitretinoin and INF‐α (*n* = 1/3). In PR, the highest response rate is seen with alitretinoin monotherapy (*n* = 3/10) and the combination of alitretinoin and ECP (*n* = 3/10). For SD, the highest response rates are shown with monotherapy of alitretinoin (*n* = 5/10), followed by the combination of alitretinoin and ECP (*n* = 2/10). PR shows the highest values using ECP (*n* = 4/12) and PUVA (*n* = 3/12). Regarding the two groups early stage and advanced stage, there was no correlation between the stages and the response rate (*p* = 0.329).

**TABLE 3:** Response rate and duration of therapy with alitretinoin

	Overall	Mycosis fungoides (MF)	Sézary syndrome (SS)	Duration of alitretinoin
	*n* = 35	*n* = 28	*n* = 7	
Complete response (CR)	3 (8.6%)	3 (10.7%)	0	29 (16‐54)
Partial response (PR)	10 (28.6%)	9 (32.1%)	1 (14.3%)	18 (3‐83)
Stable disease (SD)	10 (28.6%)	8 (28.6%)	2 (28.6%)	17 (2‐47)
Progressive disease (PD)	12 (34.3%)	8 (28.6%)	4 (57.1%)	27 (2‐80)

Abbreviations: CR, complete response; MF, mycosis fungoides; PD, progressive disease; PR, partial response; SD, stable disease; SS, Sézary syndrome.

### 3.4 Evaluation of safety

**FIGURE 3:**
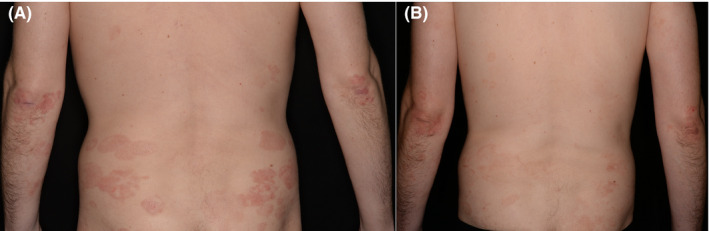
Side effects. Distribution of side effects during therapy with alitretinoin in percentages

Side effects during oral alitretinoin therapy were seen in 37.2% of patients (13/35), of whom seven patients (20.0%) developed hypertriglyceridemia, three patients (8.6%) reported mental disorders (e.g., depression, altered mood), one patient (2.9%) developed dermatitis, one patient (2.9%) noted hair loss, and one patient (2.9%) developed headache. Only three patients (8.6%) discontinued alitretinoin therapy because of side effects, all of them due to mental disorders (Figure 3).

## 4 DISCUSSION

The therapy of CTCLs should always be stage dependent with the aim of avoiding drug‐induced side effects, as there is currently no curative approach to treatment. Retinoids are considered biologic response modifiers because, in contrast to classical chemotherapy, they promote and augment an immune response without immunosuppression.^10^, ^22^ For this reason, they represent an important element in the treatment concept for all stages of CTCLs with ease of handling and a low side effect spectrum.^10^ The EORTC recommends retinoids alone or in combination with other systemic therapies for the treatment of MF and SS in stages IA–IIA as second‐line therapy, and in stage IIB as first‐line therapy.^23^ Bexarotene has been shown to be effective in clinical trials and is currently considered the only licensed retinoid for the treatment of CTCLs, however, it is not available in all countries (e.g., Canada).^18^ Furthermore, side effects are dose‐related and frequent, which may result in discontinuation of therapy.^24^ The most common side effects include hypertriglyceridemia and hypothyroidism, which occur in almost all patients, and leukopenia and neutropenia.^16^, ^25^ Another study described elevated liver enzymes in 11%, which may require discontinuation of therapy.^26^ In comparison, alitretinoin has a better safety profile with the most commonly described side effects being mild hypertriglyceridemia, headache, and cheilitis. Alhusayen et al. describe no side effects with alitretinoin in 64.3% of patients, the remaining affected patients reported hypertriglyceridemia in 40% of cases, followed by headache (13.3%) and dermatitis (13.3%), among others.^18^ This is in agreement with our study with 62.8% of the patient collective showing no side effects, 20% with hypertriglyceridemia, and 8.6% with mental disorders such as depression, which is a known adverse effect of retinoids.^27^ Mental disorders led to the discontinuation of therapy in three of our patients and has not been recorded in studies on retinoids in CTCLs so far. In our opinion, this distribution of side effect shows a clear advantage of alitretinoin compared to bexarotene. Furthermore, the treatment with bexarotene is in comparison to alitretinoin much more expensive with the monthly treatment costs of bexarotene being four times as high as those of alitretinoin (approx. 680€ vs. 2400€) in Germany. This represents an economic and financial burden for health care systems. Previous retrospective studies investigating monotherapy with alitretinoin or in combination in MF and SS described overall response rates, including CR and PR, of 90% in a study including 11 patients and 40% in a study including 40 patients.^18^, ^20^ Based on our results, we observed a similar ORR of 37.2%, whereas our patients always received additional therapy with TCS. The response rate of over 90% reported by Kapser et al. could not be confirmed in our patient population. This might be due to the larger number of subjects and the longer observation period in our study, but also due to the more advanced disease stages in our cohort.

Previous phase I/II and II/III studies from Japan and the USA showed that bexarotene can be used successfully across all disease stages. Compared to these, reporting a dose‐dependent ORR of 45%–60%, we see a similar ORR of alitretinoin, although the study designs differ greatly, thus requiring great caution in interpreting or comparing such results.^15^, ^25^, ^28^ It should be noted, that each patient in our cohort received TCS and 74.3% received further concomitant therapy, which makes a direct comparison of the different retinoid therapies difficult and bias the ORR of nearly 40% in our study compared to the ORR of 45%–60% seen for the therapy with bexarotene alone. Those additional therapies with alitretinoin include ECP, UVB, and INF‐α, which have been shown to be beneficial in previous studies.^20^ This again highlights the challenging approach in CTCLs therapy, where different therapeutic approaches are being used depending on the institution and the practitioner. This is also reflected in the 2017 EORTC guidance, where systemic therapy with retinoids is recommended alone or in combination with other systemic agents.^23^ Another promising therapeutic approach, especially for advanced stage disease, represents the targeted immunotherapy. Monoclonal antibodies against CD30 and CCR4 are current therapeutic alternatives, and as immunotherapy research progresses, additional agents are expected in the future to be developed for more targeted immunotherapy.^23^ Limitations of our study are first and foremost the retrospective and unblinded study design, in which data quality and therapy response evaluation bias are potential problems. Even though CTCLs is a rare disease, the number of patients, especially in advanced stages, is quite low. A second limitation is the evaluation of the efficacy of alitretinoin in patients that received additional topical steroids, and in the 74.3% of patients that received alitretinoin in combination with other therapies. In summary, our data show similar response rates and adverse event rates with alitretinoin in the treatment of MF and SS as reported in previous retrospective studies. Further prospective and randomized studies are necessary to precisely define the value of alitretinoin in the systemic therapy of CTCL.

## 5 CONCLUSION

Retinoids currently represent a suitable therapeutic option, especially for early stages of CTCLs. Depending on the type of retinoid administered, both the efficacy and the side effect profile differ. Although monotherapy with bexarotene shows a slightly higher ORR compared to alitretinoin alone or in combination, it is not available in all countries and leads to a fourfold higher cost burden compared to alitretinoin in Germany. In consideration of the favorable response rate observed in our and other studies to date, the lower side effects and the reduced costs, we consider alitretinoin to be a valuable therapeutic alternative to bexarotene in the treatment of MF and SS.

## CONFLICT OF INTEREST

None.

## AUTHOR CONTRIBUTIONS

All the authors made substantial contributions to the conception of the work, revised it critically, and gave final approval of the version to be published and agreed to be accountable for all aspects of the work in ensuring that questions related to the accuracy or integrity of any part of the work are appropriately investigated and resolved.

## ETHICAL APPROVAL

This study has been approved by the local ethics committee.

## Data Availability

The data that support the findings of this study are available from the corresponding author upon reasonable request.
